# Conducting a critical interpretive synthesis of the literature on access to healthcare by vulnerable groups

**DOI:** 10.1186/1471-2288-6-35

**Published:** 2006-07-26

**Authors:** Mary Dixon-Woods, Debbie Cavers, Shona Agarwal, Ellen Annandale, Antony Arthur, Janet Harvey, Ron Hsu, Savita Katbamna, Richard Olsen, Lucy Smith, Richard Riley, Alex J Sutton

**Affiliations:** 1 Department of Health Sciences, University of Leicester, 22–28 Princess Road West, Leicester LE1 6TP, UK; 2 Division of Oncology/General Practice, University of Edinburgh, Edinburgh Centre for Neuro-Oncology, Western General Hospital, Crewe Road South, Edinburgh EH4 2XU, UK; 3 Department of Health Sciences, University of Leicester, Leicester General Hospital, Leicester LE5 4PW, UK; 4 Department of Sociology, University of Leicester, Leicester LE1 7RH, UK; 5 School of Nursing, University of Nottingham, Queens Medical Centre, Nottingham NG7 2HA, UK; 6 Centre for Research in Social Policy, Loughborough University, Leicestershire LE11 3TU, UK; 7 Nuffield Research Unit, Department of Health Sciences, University of Leicester, 22–28 Princess Road West, Leicester LE1 6TP, UK

## Abstract

**Background:**

Conventional systematic review techniques have limitations when the aim of a review is to construct a critical analysis of a complex body of literature. This article offers a reflexive account of an attempt to conduct an interpretive review of the literature on access to healthcare by vulnerable groups in the UK

**Methods:**

This project involved the development and use of the method of Critical Interpretive Synthesis (CIS). This approach is sensitised to the processes of conventional systematic review methodology and draws on recent advances in methods for interpretive synthesis.

**Results:**

Many analyses of equity of access have rested on measures of utilisation of health services, but these are problematic both methodologically and conceptually. A more useful means of understanding access is offered by the synthetic construct of candidacy. Candidacy describes how people's eligibility for healthcare is determined between themselves and health services. It is a continually negotiated property of individuals, subject to multiple influences arising both from people and their social contexts and from macro-level influences on allocation of resources and configuration of services. Health services are continually constituting and seeking to define the appropriate objects of medical attention and intervention, while at the same time people are engaged in constituting and defining what they understand to be the appropriate objects of medical attention and intervention. Access represents a dynamic interplay between these simultaneous, iterative and mutually reinforcing processes. By attending to how vulnerabilities arise in relation to candidacy, the phenomenon of access can be better understood, and more appropriate recommendations made for policy, practice and future research.

**Discussion:**

By innovating with existing methods for interpretive synthesis, it was possible to produce not only new methods for conducting what we have termed critical interpretive synthesis, but also a new theoretical conceptualisation of access to healthcare. This theoretical account of access is distinct from models already extant in the literature, and is the result of combining diverse constructs and evidence into a coherent whole. Both the method and the model should be evaluated in other contexts.

## Background

Like many areas of healthcare practice and policy, the literature on access to healthcare is large, diverse, and complex. It includes empirical work using both qualitative and quantitative methods; editorial comment and theoretical work; case studies; evaluative, epidemiological, trial, descriptive, sociological, psychological, management, and economics papers, as well as policy documents and political statements. "Access" itself has not been consistently defined or operationalised across the field. There are substantial adjunct literatures, including those on quality in healthcare, priority-setting, and patient satisfaction. A review of the area would be of most benefit if it were to produce a "mid-range" theoretical account of the evidence and existing theory that is neither so abstract that it lacks empirical applicability nor so specific that its explanatory scope is limited.

In this paper, we suggest that conventional systematic review methodology is ill-suited to the challenges that conducting such a review would pose, and describe the development of a new form of review which we term "Critical Interpretive Synthesis" (CIS). This approach draws is sensitised to the range of issues involved in conducting reviews that conventional systematic review methodology has identified, but draws on a distinctive tradition of qualitative inquiry, including recent interpretive approaches to review [1]. We suggest that that using CIS to synthesise a diverse body of evidence enables the generation of theory with strong explanatory power. We illustrate this briefly using an example based on synthesis of the literature on access to healthcare in the UK by socio-economically disadvantaged people.

### Aggregative and interpretive reviews

Conventional systematic review developed as a specific methodology for searching for, appraising, and synthesising findings of primary studies [2]. It offers a way of systematising, rationalising, and making more explicit the processes of review, and has demonstrated considerable benefits in synthesising certain forms of evidence where the aim is to *test *theories, perhaps especially about "what works". It is more limited when the aim, as here, is to include many different forms of evidence with the aim of *generating *theory [3]. Conventional systematic review methods are thus better suited to the production of *aggregative *rather than *interpretive *syntheses.

This distinction between aggregative and interpretive syntheses, noted by Noblit and Hare in their ground-breaking book on meta-ethnography[1] allows a useful (though necessarily crude) categorisation of two principal approaches to conducting reviews [4]. Aggregative reviews are concerned with assembling and pooling data, may use techniques such as meta-analysis, and require a basic comparability between phenomena so that the data can be *aggregated *for analysis. Their defining characteristics are a focus on *summarising data*, and an assumption that the concepts (or variables) under which those data are to be summarised are largely secure and well specified. Key concepts are defined at an early stage in the review and form the categories under which the data from empirical studies are to be summarised.

*Interpretive *reviews, by contrast, see the essential tasks of synthesis as involving both induction and interpretation. Their primary concern is with the development of concepts and theories that integrate those concepts. An interpretive review will therefore avoid specifying concepts in advance of the synthesis. The interpretive analysis that yields the synthesis is conceptual in process and output. The product of the synthesis is not aggregations of data, but theory grounded in the studies included in the review. Although there is a tendency at present to conduct interpretive synthesis only of qualitative studies, it should in principle be possible and indeed desirable to conduct interpretive syntheses of all forms of evidence, since theory-building need not be based only on one form of evidence. Indeed, Glaser and Strauss [5] in their seminal text, included an (often forgotten) chapter on the use of quantitative data for theory-building.

Recent years have seen the emergence of a range of methods that draw on a more interpretive tradition, but these also have limitations when attempting a synthesis of a large and complex body of evidence. In general, the use to date of interpretive approaches to synthesis has been confined to the synthesis of qualitative research only [6-8]. Meta-ethnography, an approach in which there has been recent significant activity and innovation, has similarly been used solely to synthesise qualitative studies, and has typically been used only with small samples [9-11]. Few approaches have attempted to apply an interpretive approach to the whole corpus of evidence (regardless of study type) included in a review, and few have treated the literature they examine as itself an object of scrutiny, for example by questioning the ways in which the literature constructs its problematics, the nature of the assumptions on the literature draw, or what has influenced proposed solutions.

In this paper we offer a reflexive account of our attempt to conduct an interpretive synthesis of all types of evidence relevant to access to National Health Service (NHS) healthcare in the UK by potentially vulnerable groups. These groups had been defined at the outset by the funders of the project (the UK Department of Health Service Delivery and Organisation R&D Programme) as children, older people, members of minority ethnicities, men/women, and socio-economically disadvantaged people. We explain in particular our development of Critical Interpretive Synthesis as a method for conducting this review.

## Methods

### Formulating the review question

Conventional systematic review methodology [12,13] emphasises the need for review questions to be precisely formulated. A tightly focused research question allows the parameters of the review to be identified and the study selection criteria to be defined in advance, and in turn limits the amount of evidence required to address the review question.

This strategy is successful where the phenomenon of interest, the populations, interventions, and outcomes are all well specified – i.e. if the aim of the review is *aggregative*. For our project, it was neither possible nor desirable to specify in advance the precise review question, *a priori *definitions, or categories under which the data could be summarised, since one of its aims was to allow the definition of the phenomenon of access to emerge from our analysis of the literature [14]. This is not to say that we did not have a review question, only that it was not a specific hypothesis. Instead it was, as Greenhalgh and colleagues [15] describe, tentative, fuzzy and contested at the outset of the project. It did include a focus on equity and on how access, particularly for potentially vulnerable groups, can best be understood in the NHS, a health care system that is, unlike most in the world, free at the point of use.

The approach we used to further specify the review question was highly iterative, modifying the question in response to search results and findings from retrieved items. It treated, as Eakin and Mykhalovskiy [16] suggest, the question as a compass rather than an anchor, and as something that would not finally be settled until the end of the review. In the process of refining the question, we benefited from the multidisciplinary nature of our review team: this allowed a range of perspectives to be incorporated into the process, something that was also helpful and important in other elements of the review.

### Searching the literature

A defining characteristic of conventional systematic review methodology is its use of explicit searching strategies, and its requirement that reviewers be able to give a clear account of how they searched for relevant evidence, such that the search methods can be reproduced. [2] Searching normally involves a range of strategies, but relies heavily on electronic bibliographic databases.

We piloted the use of a highly structured search strategy using protocol-driven searches across a range of electronic databases but, like Greenhalgh and Peacock [17] found that was this unsatisfactory. In particular, it risked missing relevant materials by failing to pick up papers that, while not ostensibly about "access", were nonetheless important to the aim of the review. We then developed a more organic process that fitted better with the emergent and exploratory nature of the review questions. This combined a number of strategies, including searching of electronic databases; searching websites; reference chaining; and contacts with experts. Crucially, we also used expertise within the team to identify relevant literature from adjacent fields not immediately or obviously relevant to the question of "access".

However, searching generated thousands of potentially relevant items – at one stage over 100,000 records. A literature of this size would clearly be unmanageable, and well exceed the capacity of the review team. We therefore redefined the aim of the searching phase. Rather than aiming for comprehensive identification and inclusion of all relevant literature, as would be required under conventional systematic review methodology, we saw the purpose of the searching phase as identifying potentially relevant papers to provide a sampling frame. Our sampling frame eventually totalled approximately 1,200 records.

### Sampling

Conventional systematic review methodology limits the number of papers to be included in a review by having tightly specified inclusion criteria for papers. Effectively, this strategy constructs the field to be known as having specific boundaries, defined as research that has specifically addressed the review question, used particular study designs and fulfilled the procedural requirements for the proper execution of these. Interpretive reviews might construct the field to be known rather differently, seeing the boundaries as more diffuse and ill-defined, as potentially overlapping with other fields, and as shifting as the review progresses. Nonetheless, there is a need to limit the number of papers to be included in an interpretive synthesis not least for practical reasons, including the time available. Sampling is also warranted theoretically, in that the focus in interpretive synthesis is on the development of concepts and theory rather than on exhaustive summary of all data. A number of authors [18-20] suggest drawing on the sampling techniques of primary qualitative research, including principles of theoretical sampling and theoretical saturation, when conducting a synthesis of qualitative literature.

For purposes of our synthesis, we used purposive sampling initially to select papers that were clearly concerned with aspects of access to healthcare, partly informed by an earlier scoping study [21] and later used theoretical sampling to add, test and elaborate the emerging analysis. Sampling therefore involved a constant dialectic process conducted concurrently with theory generation.

### Determination of quality

Conventional systematic review methodology uses assessment of study quality in a number of ways. First, as indicated above, studies included in a review may be limited to particular study designs, often using a "hierarchy of evidence" approach that sees some designs (e.g. randomized controlled trials) as being more robust than others (e.g. case-control studies). Second, it is usual to devise broad inclusion criteria – for example adequate randomisation for RCTs – and to exclude studies that fail to meet these. Third, an appraisal of included studies, perhaps using a structured quality checklist, may be undertaken to allow sensitivity analyses aimed at assessing the effects of weaker papers.

Using this approach when confronted with a complex literature, including qualitative research, poses several challenges. No hierarchy of study designs exists for qualitative research. How or whether to appraise papers for inclusion in an interpretive reviews has received a great deal of attention, but there is little sign of an emergent consensus [22]. Some argue that formal appraisals of quality may not be necessary, and some argue that there is a risk of discounting important studies for the sake of "surface mistakes" [23]. Others propose that weak papers should be excluded from the review altogether, and several published syntheses of qualitative research have indeed used quality criteria to make decisions about excluding papers. [10,24]

We aimed to prioritise papers that appeared to be relevant, rather than particular study types or papers that met particular methodological standards. We might therefore be said to be prioritising "signal" (likely relevance) over "noise" (the inverse of methodological quality) [25]. We felt it important, for purposes of an interpretive review, that a low threshold be applied to maximise the inclusion and contribution of a wide variety of papers at the level of *concepts*. We therefore took a two-pronged approach to quality. First, we decided that only papers that were deemed to be fatally flawed would be excluded. Second, once in the review, the synthesis itself crucially involved judgements and interpretations of credibility and contribution, as we discuss later.

To identify fatally flawed papers, we used the criteria in Table 1, adapted from those proposed (at the time of our review) by the National Health Service (NHS) National Electronic Library for Health for the evaluation of qualitative research, to inform judgements on the quality of the papers. These criteria were used for assessing *all *empirical papers (but not those classified as 'reviews') regardless of study type. The final judgement about inclusion of the review rested both on an assessment of relevance as well as on the assessment of the quality of the individual papers. Decisions about relevance and quality were recorded, and a small sample of decisions about relevance and quality was reviewed. In the event, very few papers – approximately 20 – were excluded on grounds of being "fatally flawed", because even weak papers were often judged to have potentially high relevance. The value of deferring judgements of credibility and contribution until the synthesis became increasingly evident.

**Table 1:** Appraisal prompts for informing judgements about quality of papers

Are the aims and objectives of the research clearly stated? Is the research design clearly specified and appropriate for the aims and objectives of the research? Do the researchers provide a clear account of the process by which their findings we reproduced? Do the researchers display enough data to support their interpretations and conclusions? Is the method of analysis appropriate and adequately explicated?

Most fundamentally, as the review progressed, we became increasingly convinced that the assumption that all studies deemed to have satisfactorily fulfilled criteria of execution and reporting can contribute equally to a synthesis is flawed. As we discuss further below, one of the distinctive characteristics of a *critical *interpretive synthesis is its emphasis not only on summary of data reported in the literature but also on a more fundamental critique, which may involve questioning taken-for-granted assumptions.

### Data extraction

A data-extraction pro-forma was initially devised to assist in systematically identifying characteristics of research participants, methods of data collection, methods of data analysis and major findings of each paper. For both qualitative and quantitative papers, this involved extracting the titles of the categories and sub-categories using the terms used in the paper itself and a summary of the relevant material. Practically, however, it proved impossible to conduct this form of data extraction on all documents included in the review, including very large documents. We therefore summarised some documents more informally, for example using highlighter pen. More generally, the value of formal data extraction for purposes of this type of study will require further evaluation.

### Conducting an interpretive synthesis

We had intended, at the outset of this project, to use meta-ethnography, a method for interpretive synthesis where there is currently an active programme of methodological research, [9-11] as our approach to synthesis. However, this had previously only been used to synthesise qualitative studies. Our experiences of working with a large sample of papers using multiple methods led us to refine and respecify some of the concepts and techniques of meta-ethnography in order to enable synthesis of a very large and methodologically diverse literature. Eventually we had made so many amendments and additions to the original methodology that we felt it was more appropriate, helpful and informative to deem it a new methodology with its own title and processes. It is this approach which we term critical interpretive synthesis (CIS). It is important to emphasise, however, that CIS is an approach to review and is not solely a method for synthesis.

Meta-ethnography, as originally proposed [1], involves three major strategies.:

1. **Reciprocal translational analysis **(RTA). The key metaphors, themes, or concepts in each study report are identified. An attempt is then made to translate the concepts into each other. Judgements about the ability of the concept of one study to capture concepts from others are based on attributes of the themes themselves, and the concept that is "most adequate" is chosen.

2. **Refutational synthesis. **Contradictions between the study reports are characterised, and an attempt made to explain them.

3. **Lines-of-argument synthesis **(LOA) involves building a general interpretation grounded in the findings of the separate studies. The themes or categories that are most powerful in representing the entire dataset are identified by constant comparisons between individual accounts.

#### Reciprocal translational analysis

Reciprocal translational analysis involves translating findings of one paper into another by systematically comparing findings from each study, using techniques such as maps. [9] We encountered considerable methodological and practical problems in trying to apply RTA across a large set of papers, in part because of the kinds of iterations we were conducting in refining the sample. These meant that there were difficulties in identifying a stable "set" of papers on which an RTA could be conducted. RTA appears to be most suitable for a well-defined, relatively small (fewer than 50) and complete set of papers, because substitution or deletion of papers causes problems with both identifying index concepts and showing which concepts from other papers translate into these. A further problem is that, when confronted with a very large and diverse literature such as ours, RTA tends to provide only a summary in terms that have already been used in the literature. Although this may be a useful strategy as a stage on the way to a more interpretive synthesis, its value may be more limited than is the case for smaller samples of qualitative study reports where its benefits have been more evident.

Before our review, RTA had previously only been used for synthesising interpretive research, not a large and diverse body of literature, so this may be one reason why it was unsuccessful for our purposes. It is important to distinguish between the doubtful value of RTA in our synthesis (particularly because of the size and diversity of the literature), and the doubtful use of RTA in general. The diversity of the literature would also have prevented us from undertaking an aggregative synthesis using meta-analysis, but this clearly could not be read as a criticism of meta-analysis itself, but of its limitations when applying it to a diverse literature.

#### Lines of argument synthesis

Recent work [9-11] has innovated in the methodology of lines-of-argument (LOA) synthesis originally proposed by Noblit and Hare by building on Schutz's [26] notions of "orders" of constructs Schutz used the idea of "first order construct" to refer to the everyday understandings of ordinary people and "second order construct" to refer to the constructs of the social sciences. The explanations and theories used by authors in primary study reports could therefore be seen as second order interpretations. This recent work uses LOA synthesis to develop what are referred to as "third order" interpretations, which build on the explanations and interpretations of the constituent studies, and are simultaneously consistent with the original results while extending beyond them. Our experiences have led us to respecify some of this approach.

We suggest that the appropriate way of conceptualising the output of an LOA synthesis is as a *synthesising argument*. This argument integrates evidence from across the studies in the review into a coherent theoretical framework comprising a network of constructs and the relationships between them. Its function is to provide more insightful, formalised, and generalisable ways of understanding a phenomenon. A synthesising argument can be generated through detailed analysis of the evidence included in a review, analogous to the analysis undertaken in primary qualitative research. It may require the generation of what we call *synthetic constructs*, which are the result of a transformation of the underlying evidence into a new conceptual form. Synthetic constructs are grounded in the evidence, but result from an interpretation of the whole of that evidence, and allow the possibility of several disparate aspects of a phenomenon being unified in a more useful and explanatory way.

What we have called a "synthetic construct" might also be seen as a "third order construct". We suggest that the term "synthetic construct" is a more useful term because it is more explicit, and also because we emphasise that a *synthesising argument *need not consist solely of synthetic constructs. Instead, synthesising arguments may explicitly link not only synthetic constructs, but also second order constructs already reported in the literature. In effect, therefore, our approach does not make this precise distinction between second and third order constructs.

#### Refutational syntheses

We further suggest that what Noblit and Hare [1] call "refutational syntheses" are best conducted as part of the analysis that produces the synthesising argument Few published meta-ethnographies have in fact reported a separate refutational synthesis. It is, we suggest, more productive instead to adopt a critical and reflexive approach to the literature, including consideration of contradictions and flaws in evidence and theory.

An important element of producing a synthesising argument is the need, when conducting the analysis, to consider and reflect on the credibility of the evidence, to make critical judgements about how it contributes to the development of the synthesising argument, and to root the synthesising argument appropriately in critique of existing evidence. Clearly, credibility depends on the quality of the research, its currency, and the robustness of its theoretical base. But more generally, a critical interpretive synthesis is critical in the broader sense of *critique *rather than this more limited sense of critical appraisal, in which each study is judged against the standards of its type. Critique may involve identification of the research traditions or meta-narratives that have guided particular fields of research [27] as well as critical analysis of particular forms of discourses. Its aim is therefore to treat the literature as warranting critical scrutiny in its own right.

### Conducting the analysis

Our analysis of the evidence, in order to produce a synthesising argument, was similar to that undertaken in primary qualitative research. We began with detailed inspection of the papers, gradually identifying recurring themes and developing a critique. We then generated themes that helped to explain the phenomena being described in the literature, constantly comparing the theoretical structures we were developing against the data in the papers, and attempting to specify the categories of our analysis and the relationships between them. To facilitate the process of identifying patterns, themes, and categories across the large volumes of text-based data in our study, we used QSR N5 software. However, it is important to note that, as with any qualitative analysis, full transparency is not possible because of the creative, interpretive processes involved. Nonetheless, the large multidisciplinary team involved in the review, and the continual dialogue made necessary by this, helped to introduce "checks and balances" that guarded against framing of the analysis according to a single perspective.

A key feature of this process that distinguishes it from some other current approaches to interpretive synthesis (and indeed of much primary qualitative research) was its aim of being *critical*: its questioning of the ways in which the literature had constructed the problematics of access, the nature of the assumptions on which it drew, and what has influenced its choice of proposed solutions. Our critique of the literature was thus dynamic, recursive and reflexive, and, rather than being a stage in which individual papers are excluded or weighted, it formed a key part of the synthesis, informing the sampling and selection of material and playing a key role in theory generation.

### Findings: access to healthcare by socio-economically disadvantaged people

Our critical interpretive synthesis of the literature on access to healthcare by socio-economically disadvantaged people in the UK included 119 papers. Early analytic categories were tentative and contingent, but gradually became firmed up and more highly specified as our analysis continued. Our synthesis involved a critique of the tendency to use measures of utilisation as a means of assessing the extent to which access to healthcare is equitable. It further involved the generation of a synthesising argument that has the synthetic construct of *candidacy *at its core. For space reasons, we can report here only a brief illustrative summary.

### Critique of utilisation as a measure of access

Much of the evidence on whether access to healthcare in the UK is equitable has relied on measuring utilisation of health services. This approach measures the units of healthcare (consultations, procedures, etc) that people have actually consumed. The literature suggests that different groups have identifiable patterns of use of services, but the significance of these is often difficult to interpret. General practice (GP) consultation rates among socio-economically disadvantaged people have generally been found to be higher [28,29] though some recent work has suggested that social class variables are generally insignificant in explaining health service use [30] Studies that have attempted to adjust for need, usually on the basis of estimates of morbidity, have generally suggested that the apparent excess of GP consultation can be explained by higher need [31].

Our critique of the literature suggests that utilisation is a generally unhelpful measure of equity of access. Not only do the logistical and practical problems of conducting utilisation studies pose substantial threats to validity and reliability, these studies are problematic for other reasons. They rely on a largely untested set of normative (i.e. ideas about how the world ought to be) and somewhat questionable assumptions about the "correct" level of utilisation, and on a difficult-to-measure (or conceputalise) estimates of "need". They often invoke normative assumptions about need relative to some apparently privileged though often ill-defined reference group (such as "affluent" people), and therefore risk failing to identify problems in access for that reference group. Misleadingly reassuring results may be produced that indicate that "need" and use or receipt are proportionate. We argue that utilisation, or, more appropriately, *receipt *of healthcare is the outcome of many different complex processes, which all need to be recognised if access is to be properly understood.

Our analysis suggested that a focus instead on *candidacy*, a synthetic construct that we generated during the course of our analysis, would demonstrate the vulnerabilities associated with socio-economic disadvantage, emphasise the highly dynamic, multi-dimensional and contingent character of access, and allow a more insightful interpretation of the evidence on receipt of healthcare.

### Candidacy

Our synthesising argument around access to healthcare by socio-economically disadvantaged people is organised around a set of central concepts and, in particular, the core synthetic category of "candidacy". Candidacy functions as a synthetic construct because it is the product of the transformation of the evidence into a new conceptual form. It is distinct from earlier uses of the term "candidacy", including its use in the lay epidemiology of heart disease [32].

We have defined candidacy as follows: candidacy describes the ways in which people's eligibility for medical attention and intervention is jointly negotiated between individuals and health services. Our synthesising argument runs as follows: candidacy is a dynamic and contingent process, constantly being defined and redefined through interactions between individuals and professionals, including how "cases" are constructed. Accomplishing access to healthcare requires considerable work on the part of users, and the amount, difficulty, and complexity of that work may operate as barriers to receipt of care. The social patterning of perceptions of health and health services, and a lack of alignment between the priorities and competencies of disadvantaged people and the organisation of health services, conspire to create vulnerabilities. Candidacy is managed in the context of operating conditions that are influenced by individuals, the setting and environment in which care takes place, situated activity, the dynamics of face-to-face activity, and aspects of self (such as gender), the typifications staff use in categorising people and diseases, availability of economic and other resources such as time, local pressures, and policy imperatives.

### Identification of candidacy

How people recognise their symptoms as needing medical attention or intervention is clearly key to understanding how they assert a claim to candidacy. Our analysis suggests that people in more deprived circumstances are likely to manage health and to recognise candidacy as a series of crises. There is significant evidence of lower use of preventive services among more deprived groups, [33,34] as well as evidence of higher use of accident and emergency facilities, emergency admissions and out-of-hours use [35,35,37,38]. Among more deprived groups, there is a tendency to seek help in response to specific events that are seen as warranting candidacy. "Warning signs" may be downgraded in importance by socio-economically disadvantaged populations because of a lack of a positive conceptualisation of health, [39,40] the normalisation of symptoms within deprived communities [41-43], and fear of being "blamed" by health professionals [44].

### Navigation

Using services requires considerable work on the part of people. First, people must be aware of the services on offer, and there has been persistent concern that more deprived people may lack awareness of some services [45,46]. Second, using health services requires the mobilisation of a range of practical resources that may be variably available in the population. A key practical resource that impacts on the ability to seek care for the socio-economically disadvantaged, for example, is transport [44,47,48]. Other practical resources that may impact on the ability of disadvantaged groups to negotiate health services include more rigid patterns of working life [47]. Goddard and Smith [49] summarise evidence suggesting that those from more deprived social groups face financial costs of attending health services which, though not sufficient to dissuade them from using services when they are ill (i.e. in response to a specific "event"), act as a barrier to attending "optional" services related to health promotion and health prevention

### The permeability of services

Patterns of use of health services reflect issues in the organisation of services as much as they reflect a tendency to manage health as a series of crises on the part of disadvantaged people. We generated the synthetic construct of "permeability" to refer to the ease with which people can use services. Porous services require few qualifications of candidacy to use them, and may require the mobilisation of relatively fewer resources. Such services might include Accident and Emergency departments. Services that are less permeable demand qualifications (such as a referral), and also demand a higher degree of cultural alignment between themselves and their users, particularly in respect of the extent to which people feel comfortable with the organisational values of the service. Such services might include out-patients clinics in hospitals.

Services that are less permeable tend to have high levels of default by socio-economically disadvantaged people [50-53]. Appointments systems, for example, are a threat to permeability by socio-economically disadvantaged people because they require resources and competencies (including stable addresses, being able to read, and being able to present in particular places at particular times [33,50,54] In addition, the extent to which people feel alienated from the cultural values of health services and their satisfaction with services have important implications for which services they choose to use [41,55].

### Appearances at health services

*Appearing *at health services involves people in asserting a claim to candidacy for medical attention or intervention. Whatever the nature of the claim, making it clearly involves work that requires a set of competencies, including the ability to formulate and articulate the issue for which help is being sought, and the ability to present credibly. More deprived people are at risk in these situations: they may be less used to or less able to provide coherent abstracted explanations of need, and may feel intimidated by their social distance from health professionals. Sword [56] points out that people with low incomes may feel alienated by the power relations that often characterise encounters with professionals. Dixon et al [57] and, in the US, Cooper and Roter [58] suggest that middle class people may be more adept at using their "voice" to demand better and extensive services: they may be more articulate, more confident, and more persistent, while people from lower class backgrounds are less verbally active. Somerset et al [59] report that in making referral decisions, patients' social status and their ability to articulate verbally act as background (and unexpressed) influences that affect the likelihood of referral.

### Adjudications

Once a patient has asserted their candidacy by presenting to health services, the professional judgements made about that candidacy strongly influence subsequent access to attention and interventions. We generated the synthetic construct of "adjudication" to refer to the judgements and decisions made by professionals which allow or inhibit continued progression of candidacy. May et al's [60] analysis suggests doctors' practices are often exercised through a repertoire of routine judgements about the possibilities presented by individual patients and the routinely available means of solving these. These typifications are, we suggest, strongly influenced by local conditions, including the operating conditions in which practitioners work and sensitivity to resource constraints. Candidacy of socially disadvantaged people appears to be at risk of being judged to be less eligible, at least for some types of interventions, although the evidence that this happens is not particularly strong.

Our analysis suggests that it is likely that professionals' perceptions of patients who are likely to "do well" as a result of interventions may disadvantage people in more deprived circumstances. As Hughes and Griffiths [61] identify, clinical decisions may rest on often implicit social criteria about which patients "ought" to receive care People in disadvantaged groups are more likely to smoke, to be overweight and to have co-morbidities, and professional perceptions of the cultural and health capital required to *convert *a unit of health provision into a given unit of health gain may function as barriers to healthcare [34]. In addition, perceptions of social "deservingness" may play a role [61,62]. Goddard and Smith [49] summarise evidence suggesting that independent of the severity of the disease, some GPs are more likely to refer the economically active and those with dependants. Clearly, there is potential for socially disadvantaged people to be disfavoured in such decisions.

### Offers and resistance

Much of the work on utilisation of healthcare explicitly or implicitly assumes that non-utilisation is a direct reflection of non-offer. However, this type of normative analysis fails to acknowledge that people may choose to refuse offers. There is some evidence of patterns of resistance to offers. Referral implies that a GP has identified particular features of candidacy and is seeking to match those to a service that deals with that form of candidacy, but patients can resist being referred [42,63] and can resist offers of medication [64,65].

### Operating conditions and the local production of candidacy

A small body of recent research has identified what might be called local influences on the production of candidacy, and in our analysis these are hugely important. These are the contingent and locally specific influences on interactions between practitioners and patients, which may be emergent over time through repeated encounters. Crucial to the local production of candidacy is the perceived or actual availability and suitability of resources to address that candidacy [60,63].

## Discussion

Demands from health policy-makers and managers for syntheses of evidence that are useful, rigorous and relevant are fuelling interest in the development of methods that can allow the integration of diverse types of evidence [66]. With the diversity of techniques for evidence synthesis now beginning to appear, those using existing, 'new' or evolving techniques need to produce critical reflexive accounts of their experiences of using the methods [3]. Our experience of conducting a review of access to healthcare, where there is a large, amorphous and complex body of literature, and a need to assemble the findings into a form that is useful in informing policy and that is empirically and theoretically grounded [67], has led us to propose a new method – Critical Interpretive Synthesis – which is sensitised to the kinds of processes involved in conventional systematic review while drawing on a distinctively qualitative tradition of inquiry.

Conventional systematic review methodology is well-suited to aggregative syntheses, where what is required is a summary of the findings of the literature under a set of categories which are largely pre-specified, secure, and well-defined. It has been important in drawing attention to the weaknesses of informal reviews, including perceived failures in their procedural specification and the possibility that the (thus) undisciplined reviewer might be chaotic or negligent in identifying the relevant evidence, or might construct idiosyncratic theories and marshall the evidence in support of these. It has thus revealed some of the pitfalls of informal literature review. Conventional systematic review methodology has demonstrated considerable benefits in synthesising certain forms of evidence where the aim is to *test *theories (in the form of hypotheses), perhaps especially about "what works". However, this approach is limited when the aim, confronted with a complex body of evidence, is to *generate *theory [15,27].

Current methods for conducting an interpretive synthesis of the literature, (such as meta-ethnography) are also limited, in part because application of many interpretive methods for synthesis has remained confined to studies reporting qualitative research. Realist synthesis [68], which does include diverse forms of evidence, is oriented towards theory evaluation, in particular by focusing on theories of change. Methods for including qualitative and quantitative evidence in systematic reviews developed by the EPPI Centre at the Institute of Education, London, have involved refinements and extensions of conventional systematic review methodology [6-8], and have limited their application of interpretive techniques to synthesis of qualitative evidence.

More generally, many current approaches fail to be sufficiently *critical*, in the sense of offering a critique. There is rarely an attempt to reconceptualise the phenomenon of interest, to provide a more sweeping critique of the ways in which the literature in the area have chosen to represent it, or to question the epistemological and normative assumptions of the literature. With notable exceptions such as the recent approach of meta-narrative analysis [15], critique of papers in current approaches to review tends to be limited to appraisal of the methodological specificities of the individual papers.

Conducting an interpretive review of the literature on access to healthcare by vulnerable groups in the UK therefore required methodological innovation that would be alert to the issues raised by systematic review methodology but also move beyond both its limitations and those of other current interpretive methods. The methods for review that we developed in this project (Table 2) built on conventional systematic review methodology in their sensitivity to the need for attentiveness to a range of methodological processes. Crucially, in doing so, we drew explicitly on traditions of qualitative research inquiry, and in particular on the principles of grounded theory [5].

**Table 2:** Key Processes in critical interpretive synthesis

▪ A review question should be formulated at the outset, but should remain open to modification. Precise definitions of many constructs may be deferred until late in the review and may be a product of the review itself. ▪ Searching, sampling, critique and analysis proceed hand in hand, and should be seen as dynamic and mutually informative processes. ▪ Searching initially should use a broadly defined strategy, including purposive selection of material likely or known to be relevant. ▪ The analysis should be aimed towards the development of a synthesising argument: a critically informed integration of evidence from across the studies in the review. The synthesising argument takes the form of a coherent theoretical framework comprising a network of constructs and the relationships between them. The synthesising argument links synthetic constructs (new constructs generated through synthesis) and existing constructs in the literature. ▪ There is a need for constant reflexivity to inform the emerging theoretical notions, as these guide the other processes. ▪ Ongoing selection of potentially relevant literature should informed by the emerging theoretical framework. Literatures not directly or obviously relevant to the question under review may be accessed as part of this process. ▪ CIS encourages an ongoing critical orientation to the material to be included in the review. Some limited formal appraisal of methodological quality of individual papers is likely to be appropriate. Generally the aim will be to maximise relevance and theoretical contribution of the included papers. ▪ Formal data extraction procedures may be helpful, particularly at the outset of the review, but are unlikely to be an essential feature of the approach. ▪ CIS does not offer aim to offer a series of pre-specified procedures for the conduct of review. It explicitly acknowledges the "authorial voice"; that some aspects of its production of the account of the evidence will not be visible or auditable; and that its account may not be strictly reproducible. Its aim is to offer a theoretically sound and useful account that is demonstrably grounded in the evidence. ▪ CIS demands constant reflexivity on the part of authors of reviews. Authors are charged with making conscientious and thorough searches, with making fair and appropriate selections of materials, with seeking disconfirming evidence and other challenges to the emergent theory, and with ensuring that the theory they generate is, while critically informed, plausible given the available evidence.

In addition to its explicit orientation towards theory generation, perhaps what most distinguishes CIS from conventional systematic review methods is its rejection of a "stage" approach to review. Processes of question formulation, searching, selection, data extraction, critique and synthesis are characterised as iterative, interactive, dynamic and recursive rather than as fixed procedures to be accomplished in a pre-defined sequence. CIS recognises the need for flexibility in the conduct of review, and future work would need to assess how far formal methods of critical appraisal and data extraction will be essential elements of the method. Our experience suggests that while attention to scientific quality is required, more generally the emphasis should be on critique rather than critical appraisal, and an ongoing critical orientation to the material examined and to emerging theoretical ideas. Formal data extraction may also be an unnecessarily constraining and burdensome process.

CIS emphasises the need for theoretical categories to be generated from the available evidence and for those categories to be submitted to rigorous scrutiny as the review progresses. Further, it emphasises a need for constant reflexivity to inform the emerging theoretical notions, and guides the sampling of articles. Although CIS demands attention to flaws in study design, execution and reporting in our judgements of the quality of individual papers, its critical approach goes beyond standard approaches. Thus, in our review, some methodologically weak papers were important in terms of their theoretical contribution, or in terms of demonstrating the breadth of evidence considered in the construction of particular categories, or in terms of providing a more comprehensive summary of the evidence, while a single strong paper might be pivotal in the development of the synthesis. Hughes and Griffiths' paper on micro-rationing of healthcare [61], for example, was a key paper in helping to generate the construct of candidacy that later came to unify the themes of our analysis. The critical interpretation in our analysis focused on how a synthesising argument could be fashioned from the available evidence, given the quality of the evidence and the kinds of critiques that could be offered of the theory and assumptions that lay behind particular approaches. In treating the literature as an object of scrutiny in its own right, CIS problematises the literature in ways that are quite distinctive from most current approaches to literature reviewing.

### Access to healthcare

The CIS approaches we adopted deferred final definition of the phenomenon of access and the appropriate ways of conceptualising it until our analysis was complete. Our critique of the current literature focused on the inadequacies of studies of utilisation as a guide to explaining inequities in health care. The conceptual model of access that we developed emphasises candidacy as the core organising construct, and recasts access as highly dynamic and contingent, and subject to constant negotiation.

In this conceptual model of access to healthcare, health services are continually constituting and seeking to define the appropriate objects of medical attention and intervention, while at the same time people are engaged in constituting and defining what they understand to be the appropriate objects of medical attention and intervention. Candidacy describes how people's eligibility for healthcare is determined between themselves and health services. Candidacy is a continually negotiated property of individuals, subject to multiple influences arising both from people and their social contexts and from macro-level influences on allocation of resources and configuration of services. "Access" represents a dynamic interplay between these simultaneous, iterative and mutually reinforcing processes. By attending to how vulnerabilities arise in relation to candidacy, the phenomenon of access can be much better understood, and more appropriate recommendations made for policy, practice and future research. Although our review focused on the UK, we suggest that the construct of candidacy is transferable, and has useful explanatory value in other contexts.

In addition to the core construct of candidacy, our analysis required the production of a number of other linked synthetic constructs – constructs generated through an attempt to summarise and integrate diverse concepts and data – including "adjudications" and "offers". It was also possible to link existing "second order" constructs, for example relating to help-seeking as the identification of candidacy by patients, into the synthesising argument, and making these work as synthesising constructs. We feel that this approach allows maximum benefit to be gained from previous analyses as well as the new synthesis.

### Reflections on the method

Clearly, questions can be raised about the validity and credibility of the CIS analysis we have presented here. Conventional systematic review methodology sets great store by the reproducibility of its protocols and findings. It would certainly have been possible to produce an account of the evidence that was more reproducible. For example, we could have used the evidence to produce a thematic summary that stuck largely to the terms and concepts used in the evidence itself. However, we felt it important that we produced an interpretation of the evidence that could produce new insights and fresh ways of understanding the phenomenon of access, and that the "critical voice" of our interpretation was maintained throughout the analysis. Simply to have produced a thematic summary of what the literature was saying would have run the risk of accepting that the accounts offered in the evidence-base were the only valid way of understanding the phenomenon of access to healthcare by vulnerable groups. We therefore make no claim to reproducibility, but wish to address some possible concerns. First, it could be argued that a different team using the same set of papers would have produced a different theoretical model. However, the same would be true for qualitative researchers working with primary qualitative data, who accept that other possible interpretations might be given to, say, the same set of transcripts. Clearly, the production of a synthesizing argument, as an interpretive process, produces one privileged reading of the evidence, and, as the product of an authorial voice, it cannot be defended as an inherently reproducible process or product. We would suggest, however, that our analysis can be defended on the grounds that it is demonstrably grounded in the evidence; that it is plausible; that it offers insights that are consistent with the available evidence; and that it can generate testable hypotheses and empirically valuable questions for future research.

Second, subjecting a question to continual review and refinement, as we did, may make it more difficult for those conducting critical interpretive reviews to demonstrate, as required by conventional systematic review methodology, the "transparency", comprehensiveness, and reproducibility of search strategies. This dilemma between the "answerable" question and the "meaningful" question has received little attention, but it underpins key tensions between the two ends of the academic/pragmatic systematic review spectrum. On balance, faced with a large and amorphous body of evidence in an area such as access to healthcare, and given the aims of an interpretive synthesis, we feel that our decision not to limit the focus of the review at the outset, and our subsequent sampling strategies, were well justified. Our decision not to commit to a particular view of what access might be and how it should be assessed at the outset of the project was critical to our subsequent development of a more satisfactory understanding of access.

Third, it could be argued that we have synthesized too small a sample of the available papers, or that the processes used to select the papers are not transparent. We recognize that we have analyzed and synthesized only a fraction of all relevant papers in the area of access to healthcare by vulnerable groups. However, a common strategy in conventional systematic review is to limit the study types to be included; this strategy also might result in only a proportion of the potentially relevant literature being synthesised. While we have described our methods for sampling as purposive, it is possible that another team using the same approach could have come up with a different sample, because, particularly in the later stages of our review, our sampling was highly intuitive and guided by the emerging theory.

The final version of the conceptual model of access to healthcare that we eventually developed did not emerge until quite late in the review process, and much of the later sampling was directed at testing and purposively challenging the theory as we began to develop it. Again, such forms of searching and sampling do not lend themselves easily to reproducibility or indeed auditability. Testing whether the interpretations change in response to different findings will be an important focus for future research, which will also need to evaluate whether apparently disconfirming evidence is the result of methodological flaws or poses a genuine challenge to theory.

## Conclusion

Conducting interpretive reviews in challenging areas where there is a large body of diverse evidence demands an approach that can draw on the strengths of conventional systematic review methodology and on the recent advances in methods for interpretive synthesis. We have termed the approach we developed to this review "critical interpretive synthesis". We believe that this methodology offers the potential for insight, vividness, illumination, and reconceptualisation of research questions, particularly in challenging areas such as access to healthcare, and look forward to further evaluations of its application.

## Competing interests

The author(s) declare that they have no competing interests.

## Authors' contributions

MDW designed the project, led and supervised its execution, and drafted the manuscript. EA, AA, JH, RH, SK, RO, LS, RR and AJS participated in the design of the study. All authors engaged in searching, screening, sampling, data extraction, and critical appraisal/critique activities, and contributed to the thematic analysis. DC and SA managed the searching, maintained the databases and coded material using N6 software. All authors contributed to the draft of the manuscript.

## Pre-publication history

The pre-publication history for this paper can be accessed here:


